# Supplementary material for: A Yeast-Based High-Throughput Screening Platform for the Discovery of Novel pre-mRNA Splicing Modulators
Source: ACS Chem Biol. 2026 Jan 12;21(2):326–38. doi: 10.1021/acschembio.5c00867 (PMC12930365; doi:10.1021/acschembio.5c00867)
Supplement: Supplementary file 1 [file cb5c00867_si_001.pdf]

A Yeast-Based High-Throughput Screening Platform for the Discovery of Novel pre-mRNA Splicing Modulators

Sierra L. Love[1,2], Henrik Vollmer[2], Ya-Chu Chang[3,4], Joshua C. Paulson[2], Tucker J. Carrocci[2], Melissa S. Jurica[5], Hai Dang Nguyen[3,4], and Aaron A. Hoskins[2,6]

[1]Genetics Training Program, University of Wisconsin-Madison, Madison, WI 53706
[2]Department of Biochemistry, University of Wisconsin-Madison, Madison, WI 53706
[3]Department of Pharmacology, University of Minnesota, Minneapolis, MN 55455
[4]Masonic Cancer Center, University of Minnesota, Minneapolis, MN 55455
[5]Center for Molecular Biology of RNA, University of California-Santa Cruz, Santa Cruz, CA 95064
[6]Department of Chemistry, University of Wisconsin-Madison, Madison, WI 53706

CORRESPONDING AUTHOR:
Aaron A. Hoskins, ahoskins@wisc.edu

Supplemental Materials:

Supplemental Figures S1-S5
Supplemental Tables S1-S3

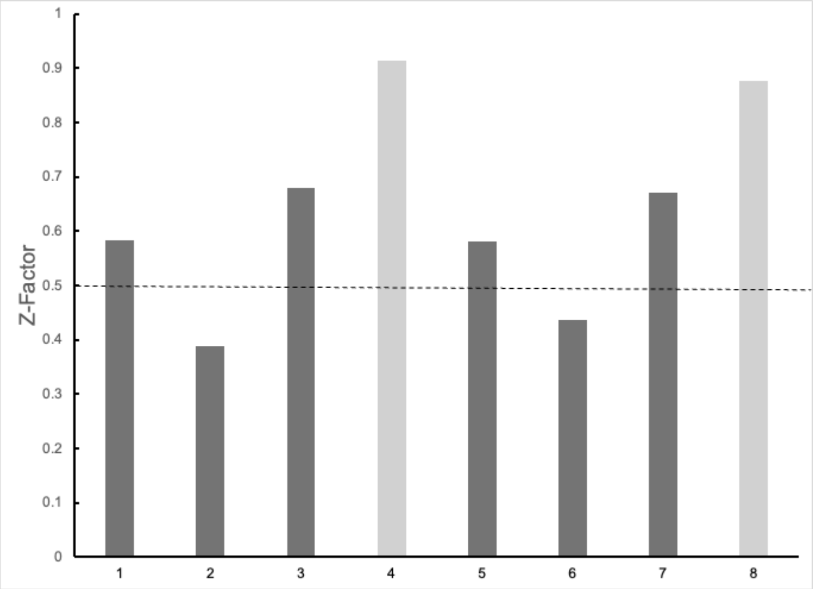

Figure S1. Z-score values for plates used in screening the FDA-compound library. Plates 1-4 and 5-8 are replicates of one another. Plates 1-3 and 5-7 used phleomycin as the negative control. Plates 4 and 8 used herboxidiene. Z scores were calculated according to the equation $Z = 1 - \frac{3(\sigma_s+\sigma_c)}{|\mu_s-\mu_s|}$. Note that the FDA-compound library contains multiple antifungals, which contributed to the lower values for plates 2 and 6.

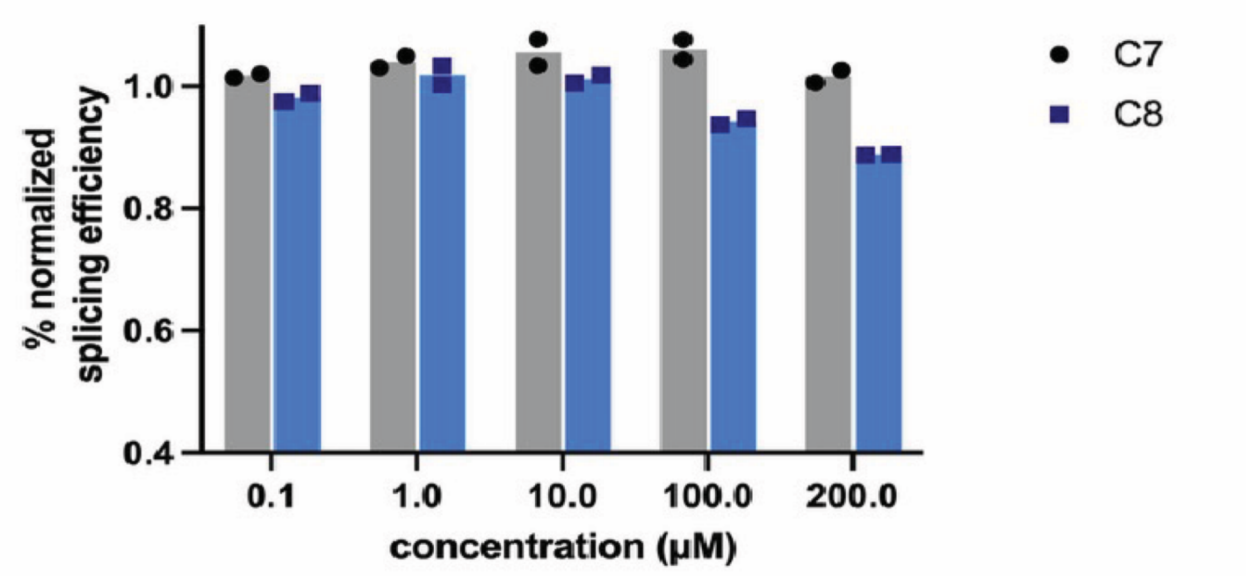

Figure S2. Compound C7 Does Not Reduce Splicing Efficiency in HeLa Nuclear Extract
In vitro splicing assay results using HeLa nuclear extracts and the AdML pre-mRNA substrate in the presence of compounds C7 and C8. Bar heights represent the averages of two technical replicates (dots, squares).

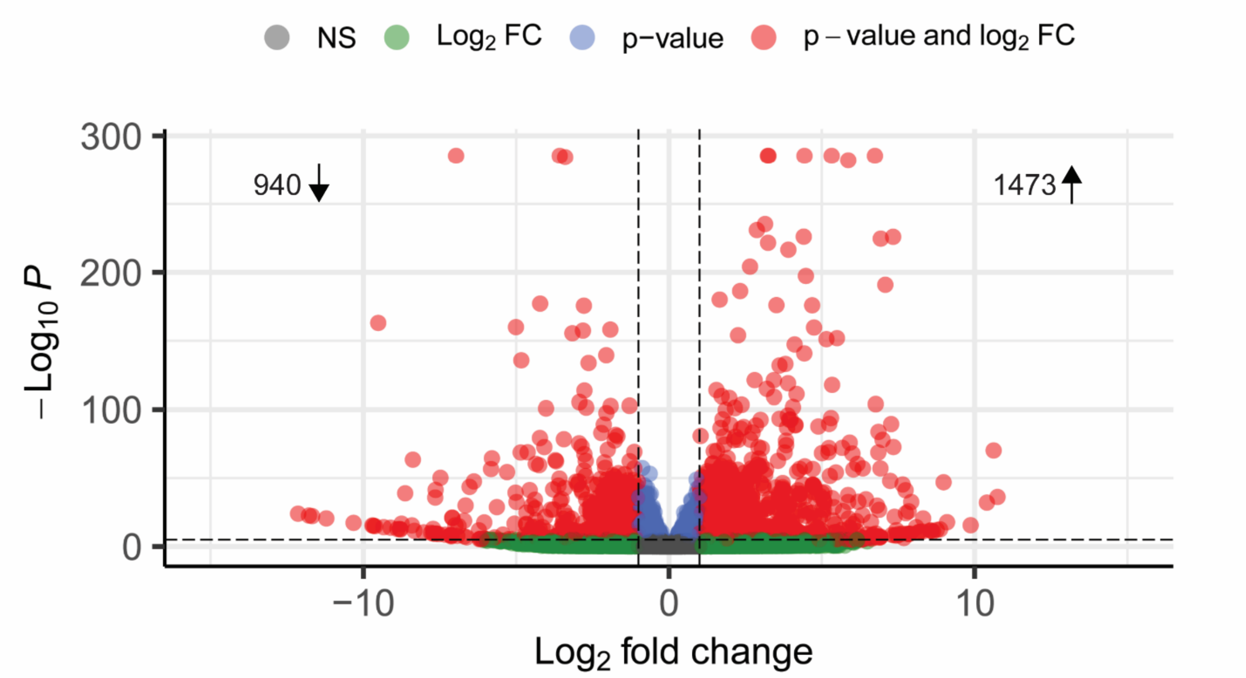

Figure S3. DEGs in P95H Mutant Cells relative to WT upon DMSO treatment
Differential gene analysis of $SRSF2^{WT/WT}$ and $SRSF2^{WT/P95H}$ mutant cells in the DMSO control. DEGs were defined as those with $\log_2$ fold change <-1 or >1 and a $-\log_{10}$ *p*-value >0.05 (red dots). Genes downregulated in the P95H cell line are shown on the left, and upregulated genes on the right.

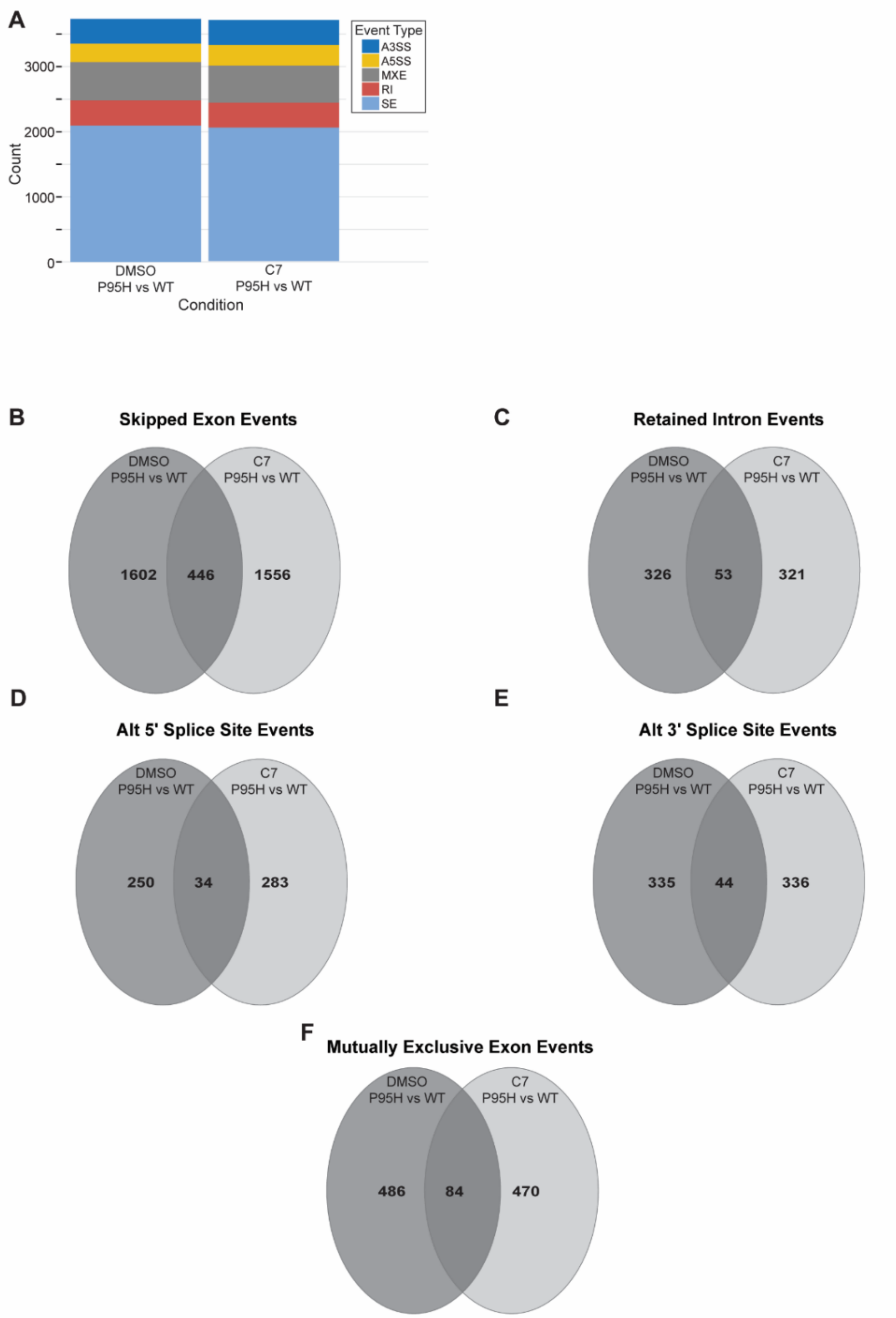

Figure S4. C7 Treatment Induces Changes in pre-mRNA Splicing (A) Stacked bar graph showing the distribution of novel alternative splicing events found in P95H mutant cells relative to WT after treatment with DMSO (left bar) and compound C7 (right bar). A3SS/A5SS: alternative 3'/5' splice sites; MXE: mutually exclusive exons; RI: retained introns; SE: skipped exons. **(B–F)** Venn diagrams showing unique splicing events in the P95H mutant cells in comparison to WT upon treatment with DMSO (dark gray) or compound C7 (light gray). Overlapping events were unique to the P95H cell line relative to WT and found upon treatment with either DMSO or C7.

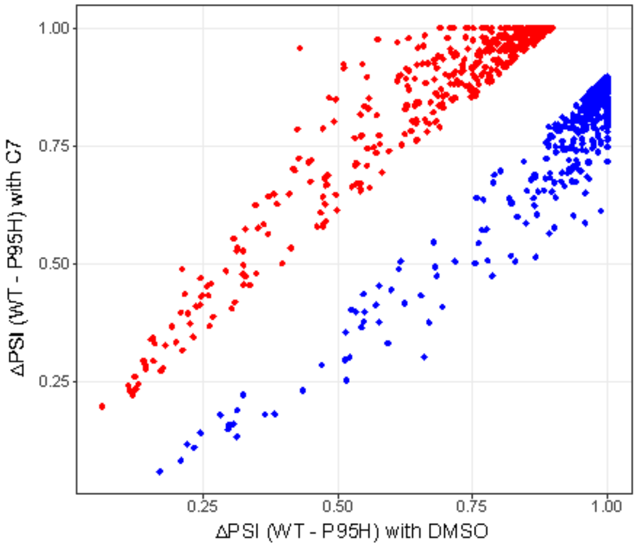

Figure S5. Treatment with C7 alters ΔPSI Arising from the P95H Mutation Relative to WT. In this plot, ΔPSI values were determined for WT vs. P95H mutant cells (averaged from the replicates) in the DMSO (x-axis) and C7-treatment (y-axis) conditions for exon skipping events. Since we used a ΔPSI threshold of >10% to identify significant events, this results in no events near the diagonal. Events above the diagonal (red) indicated that C7 treatment increases differences in PSI (ΔPSI) when comparing the WT and P95H cells. Events below the diagonal (blue) indicates the C7 treatment decreases these differences.

Table S1. Selleck FDA Pilot Screening Hits

Compound	Use/MoA	Specificity
Benazepril	Hypertension treatment/ACE inhibitor	Hsh155
Bifonazole	Antifungal	Hsh155
Butoconazole	Antifungal	Both
Caspofungin	Antifungal	Both
Climbazole	Antifungal	Both
Cyproheptadine	Antihistamine	Both
Cytidine	Antidepressant	Hs-Hsh155
Econazole	Antifungal	Both
Everolimus	Immunosuppressant	Both
Fenticonazole	Antifungal	Both
Isocinazole	Antifungal	Both
Miconazole	Antifungal	Both
Rapaycin	Antibiotic/immunosupressant	Both
Rivastigmine	cognition enhancing med	Both
Sulconazole	Antifungal	Both
Tioconazole	Antifungal	Hsh155
Voriconazole	Antifungal	Both
Amorolfine	Antifungal	Hs-Hsh155

Table S2. Secondary Screening Hits

Structure	Name	Library	Specificiy	Lipinski Rules	PAINS parameters
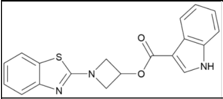	1-(1,3-benzothiazol-2-yl)azetidin-3-yl 1H-indole-3-carboxylate	Life Chemicals 4	WTBrr2, HsBrr2	Pass	Pass
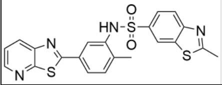	2-methyl-N-(2-methyl-5-{[1,3]thiazolo[5,4-b]pyridin-2-yl}phenyl)-1,3-benzothiazole-6-sulfonamide	Life Chemicals 4	WTHsh155	Pass	Pass
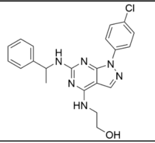	2-[[1-(4-chlorophenyl)-6-[[(1S)-1-phenylethyl]amino]pyrazolo[3,4-d]pyrimidin-4-yl]amino]ethanol	Life Chemicals 4	HsBrr2	Pass	Pass
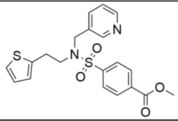	methyl 4-{[(pyridin-3-yl)methyl][2-(thiophen-2-yl)ethyl]sulfamoyl}benzoate	Life Chemicals 4	WTBrr2	Pass	Pass
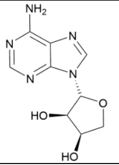	9-beta-d-erythrofuranosyladenine (EFA)	NCI:DTP	Nonspecific	Pass	Pass
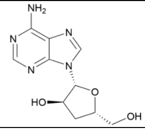	3'-Deoxyadenosine (Cordycepin)	NCI:DTP	Nonspecific	Pass	Pass
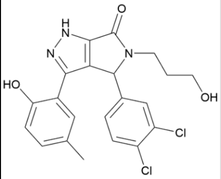	4-(3,4-dichlorophenyl)-3-(2-hydroxy-3,5-dimethylphenyl)-5-(3-hydroxypropyl)-1,4-dihydropyrrolo[3,4-c]pyrazol-6-one	NCI:NExT	HsBrr2	Pass	Pass
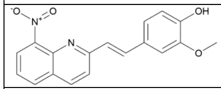	2-methoxy-4-[(E)-2-(8-nitroquinolin-2-yl)ethenyl]phenol (drug C8)	NCI:NExT	HsBrr2	Pass	Pass
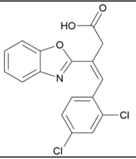	(Z)-3-(1,3-benzoxazol-2-yl)-4-(2,4-dichlorophenyl)but-3-enoic acid	NCI:NExT	HsBrr2	Pass	Pass
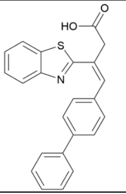	(Z)-3-(1,3-benzothiazol-2-yl)-4-(4-phenylphenyl)but-3-enoic acid	NCI:NExT	WTHsh155	Pass	Pass
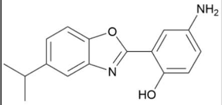	4-Amino-2-[5-(propan-2-yl)-1,3-benzoxazol-2-yl]phenol (drug C7)	NCI:NExT	HsBrr2	Pass	Pass

Table S3. GO-term Enrichment of Alternative Splicing Events Unique to P95H Mutant Cells Treated with C7 relative to WT

Alt Splicing Type	Enrichment FDR	nGenes	Fold Enrichment	Pathway
A3SS	0.00515325	10	6.45954492	GO:0002181 cytoplasmic translation
A3SS	0.01622916	20	2.9331607	GO:0006412 translation
A3SS	0.01729273	20	2.82505896	GO:0043043 peptide biosynthetic proc.
A3SS	0.01729273	22	2.63770364	GO:0043604 amide biosynthetic proc.
A3SS	0.02419751	22	2.53208988	GO:0006518 peptide metabolic proc.
A3SS	0.02441377	23	2.44411986	GO:0006396 RNA processing
A3SS	0.01729273	26	2.37448561	GO:0034645 cellular macromolecule biosynthetic proc.
A3SS	0.00515325	37	2.25331371	GO:1901566 organonitrogen compound biosynthetic proc.
A5SS	0.024031898	3	29.35970915	GO:0006287 base-excision repair gap-filling
A5SS	0.024031898	3	29.35970915	GO:1902969 mitotic DNA replication
A5SS	0.00897284	4	23.82816975	GO:0006268 DNA unwinding involved in DNA replication
A5SS	0.028084961	4	14.42231327	GO:0033260 nuclear DNA replication
A5SS	0.032995488	4	13.04875962	GO:0044786 cell cycle DNA replication
A5SS	0.023896835	5	10.87396635	GO:0042255 ribosome assembly
A5SS	0.029093228	6	7.27497218	GO:0071103 DNA conformation change
A5SS	0.00897284	8	6.683511027	GO:0006261 DNA-templated DNA replication
A5SS	0.00897284	11	4.608965555	GO:0042254 ribosome biogenesis
A5SS	0.032995488	10	3.937125749	GO:0006310 DNA recombination
A5SS	0.001823076	18	3.800023989	GO:0006281 DNA repair
A5SS	0.00897284	14	3.768502288	GO:0022613 ribonucleoprotein complex biogenesis
A5SS	0.032995488	12	3.40402425	GO:0034470 ncRNA processing
A5SS	0.021842731	15	3.186325024	GO:0034660 ncRNA metabolic proc.
A5SS	0.000614589	26	3.130326342	GO:0006259 DNA metabolic proc.
A5SS	0.003695002	23	2.912454204	GO:0006396 RNA processing
A5SS	0.008035651	21	2.900455138	GO:0006974 cellular response to DNA damage stimulus
A5SS	0.024031898	19	2.613682274	GO:0000278 mitotic cell cycle
A5SS	0.024031898	21	2.452899827	GO:0051276 chromosome organization
A5SS	0.00897284	53	1.72649423	GO:0006996 organelle organization
RI	0.00976974	64	1.67575291	Cellular protein modification process
RI	0.00976974	31	2.33850003	Cell cycle process
RI	0.00976974	64	1.67575291	Protein modification process
RI	0.01172098	26	2.51641391	Mitotic cell cycle
RI	0.01445863	36	2.0343783	Cell cycle
RI	0.01445863	65	1.61216407	Macromolecule modification
RI	0.01445863	55	1.69585565	Cellular component biogenesis
RI	0.01445863	8	6.7374021	Positive regulation of proteasomal protein catabolic process
RI	0.01445863	23	2.542198	Mitotic cell cycle process
RI	0.01752097	51	1.71957746	Cellular component assembly
RI	0.01967816	2	112.851485	Neural plate elongation
RI	0.01967816	2	112.851485	Convergent extension involved in neural plate elongation
RI	0.01967816	8	5.82459278	Positive regulation of proteolysis involved in cellular protein catabolic process
RI	0.02501625	13	3.47646755	Positive regulation of proteolysis
RI	0.04097803	8	5.1006321	Positive regulation of cellular protein catabolic process
RI	0.04143575	34	1.90042125	Phosphorylation
RI	0.04440153	3	24.1824611	CTP biosynthetic process
RI	0.04440153	16	2.7693616	Regulation of mitotic cell cycle
RI	0.04445504	3	22.570297	Fructose metabolic process
RI	0.04445504	46	1.66543738	Phosphate-containing compound metabolic process
SE	0.032711726	3	23.1902027	GO:1901483 reg. of transcription factor catabolic proc.
SE	0.026891774	25	2.321341612	GO:0048193 Golgi vesicle transport
SE	0.023895547	26	2.310135135	GO:0006310 DNA recombination
SE	0.017804515	33	2.11256505	GO:0034470 ncRNA processing
SE	0.010673076	42	2.013412948	GO:0034660 ncRNA metabolic proc.
SE	0.032711726	47	1.765799153	GO:0016071 mRNA metabolic proc.
SE	0.010811217	61	1.743194535	GO:0006396 RNA processing
SE	0.017804515	62	1.684584145	GO:0006259 DNA metabolic proc.
SE	0.010673076	87	1.574057059	GO:0046907 intracellular transport
SE	0.024212078	91	1.490330823	GO:1901566 organonitrogen compound biosynthetic proc.
SE	1.38E-05	200	1.470293403	GO:0006996 organelle organization
SE	0.017804515	103	1.46427027	GO:0071705 nitrogen compound transport
SE	0.017804515	120	1.417277476	GO:0071702 organic substance transport
SE	0.00089922	201	1.380185282	GO:0018130 heterocycle biosynthetic proc.
SE	0.001275314	199	1.363424282	GO:0019438 aromatic compound biosynthetic proc.
SE	0.001058461	206	1.362184704	GO:1901362 organic cyclic compound biosynthetic proc.
SE	0.002211429	194	1.35560055	GO:0034654 nucleobase-containing compound biosynthetic proc.
SE	0.010673076	191	1.317664351	GO:0031326 reg. of cellular biosynthetic proc.
SE	0.016957976	183	1.313160701	GO:0010556 reg. of macromolecule biosynthetic proc.
